# Effects of Different Traditional Chinese Mind–Body Exercises on Learning Abilities, Executive Functions, and Brain Connectivity in Children with Learning Difficulties

**DOI:** 10.3390/bs15030303

**Published:** 2025-03-04

**Authors:** Xiaoyan Wang, Haojie Li

**Affiliations:** 1 Department of Physical Education, Hangzhou Normal University, Hangzhou 311121, China; 20021206@hznu.edu.cn; 2 School of Exercise and Health, Shanghai University of Sport, Shanghai 200438, China

**Keywords:** learning difficulties, Tai Chi, Baduanjin, Health Qigong Yijinjing, executive function, brain connectivity, fNIRS

## Abstract

This study examines the effects of three traditional Chinese mind–body exercises—Tai Chi (TC), Baduanjin (BD), and Health Qigong Yijinjing (YJJ)—on learning abilities, executive functions, and prefrontal brain connectivity in children with learning difficulties. Seventy-two children (aged 9–11) with learning difficulties were randomly assigned to TC, BD, YJJ, or a control group (CON). Intervention groups practiced for 12 weeks (45 min, three times per week), while the control group maintained their regular physical education. Assessments included Academic Performance Ranking (APR), Pupil Rating Scale (PRS), and executive functions. Granger causality analyses were conducted on the functional near-infrared spectroscopy data to derive the effective connectivity at the brain region levels. Post-intervention, all intervention groups showed significant improvements over the control group in PRS and APR scores (*p* < 0.05), with the TC group achieving higher PRS scores than the BD group. The TC group also demonstrated superior improvements in executive functions, particularly in inhibition and working memory. Additionally, the TC group exhibited significantly enhanced effective connectivity from the left and right dorsolateral prefrontal cortex to Brodmann area 8, indicating improved brain communication. Traditional Chinese mind–body exercises, particularly Tai Chi, improve academic performance, executive functions, and prefrontal cortex connectivity in children with learning difficulties. Tai Chi demonstrates superior outcomes, supporting its potential as an effective intervention for cognitive and academic development.

## 1. Introduction

Learning Disabilities (LDs) have become a growing educational problem in the global population of school-aged children. Approximately 5–15% of children worldwide suffer from learning difficulties, including dyslexia, dyscalculia, dyspraxia, and other types of disabilities (Gillberg & Soderstrom, 2003; Shapiro & Gallico, 1993). Learning difficulties not only affect academic performance but also have a profound impact on children’s emotional development, social skills, and future careers (Kronenberger & Dunn, 2003; Kelly, 2005). Especially at school age, children with learning difficulties often experience difficulties in cognitive and emotional management, and long-term academic setbacks may lead to decreased self-esteem, increased anxiety, and social isolation (Silver, 1971). Specifically, a decline in learning ability is the most direct manifestation of children with learning difficulties, especially in the areas of language comprehension, mathematical reasoning, problem solving, and memory, and these difficulties will further limit their academic performance and daily life abilities (McDowell, 2018; Handler et al., 2011). Therefore, early identification of and effective intervention with learning difficulties can not only improve children’s academic ability but also have a crucial significance on their psychological health and social adaptability.

Executive function (EF) refers to the cognitive processes required by the brain to complete complex tasks, including core functions such as working memory, cognitive flexibility, and inhibitory control (Shemtob et al., 2021). These functions play a crucial role in the learning process of children, especially children with learning difficulties, where deficits in executive function are often one of the root causes of their learning challenges (Shemtob et al., 2021). Research has shown that children with learning difficulties usually have deficits in executive functioning, as evidenced by inattention, poor working memory, difficulty switching tasks, and poor self-control (O’Brien & Pearson, 2004). For example, children with poor executive functioning are often unable to organize information effectively or manage time efficiently during the learning process, thus affecting their learning progress and effectiveness (Shaheen, 2014). In addition, deficits in executive functioning are also closely related to children’s difficulties in emotion regulation, impulsive behaviors, and poor academic performance, which interact with each other to further exacerbate the symptoms of learning difficulties. Therefore, improving the executive functions of children with learning difficulties can not only help them progress academically but also contribute to their overall cognitive abilities and social adaptability (Diamond & Lee, 2011).

Functional connectivity (FC) of the brain refers to the interactions and synergies between different regions of the brain, which are essential for efficient information processing and cognitive functions (Fingelkurts et al., 2005). During children’s brain development, the optimization of functional connectivity is directly related to various aspects of performance, such as cognitive ability, learning ability, and emotional regulation (D’Mello & Gabrieli, 2018). The study has shown that children’s executive functions and learning abilities rely heavily on the efficient connectivity of different regions of the cerebral cortex (Bidzan-Bluma & Lipowska, 2018). However, research on the functional connectivity of the brain in children with learning difficulties is still relatively limited, and the evidence is still insufficient, especially in exploring how learning difficulties affect the structure and function of children’s brain networks (Simos et al., 2011). Nonetheless, it has been suggested that there may be some degree of abnormalities in the functional connectivity of the brains of children with learning difficulties, especially among brain regions related to cognitive control, language processing, and memory (Vellutino et al., 2004). Therefore, an in-depth study of the brain functional connectivity of children with learning difficulties not only helps to reveal the neurobiological mechanisms of their cognitive deficits but also provides a theoretical basis for individualized intervention strategies.

Currently, intervention studies for children with learning difficulties mainly focus on educational and teaching modes, cognitive training, etc. Although these methods can improve academic performance and mental health to some extent, they tend to ignore the potential impact of exercise on cognitive function (Vellutino et al., 2004; Williams et al., 2022). Physical exercise has not been given enough attention as an effective cognitive intervention in traditional education and intervention approaches. In recent years, a number of studies have begun to focus on the ameliorative effects of exercise training on children with learning difficulties, particularly in terms of enhancing attention, emotion regulation, and executive function (Yin et al., 2018). However, the vast majority of studies have focused on changes in mood with exercise and lacked focus on changes in actual functional improvements, such as aerobic exercise or motor games (Pan et al., 2016). In contrast, the potential of traditional Chinese mind–body exercise, with its deep cultural background and unique mind–body regulatory mechanisms, to improve cognitive function, regulate mood, and enhance functional brain connectivity has not been fully explored. Traditional Chinese mind–body exercises not only contribute to physical health but also promote brain self-regulation and plasticity by regulating breathing, meditation, and movement coordination (Pan et al., 2016). In particular, it has been shown to have positive effects in regulating mood, improving concentration, and enhancing cognitive flexibility and executive function (Huston & McFarlane, 2016). Therefore, intervention strategies based on traditional Chinese mind–body exercises may provide a new direction for the improvement of children with learning difficulties. This study used Tai Chi (TC), Baduanjin (BD), and Health Qigong Yijinjing from the traditional Chinese mind–body exercises for intervention training.

This study aimed to explore the effects of different types of traditional Chinese mind–body exercises on learning ability, executive function, and brain connectivity in children with learning difficulties. Unlike previous studies, this study not only focuses on the effects of traditional mind–body exercises on behavioral performance, such as attention, but also explores their deeper effects on executive function and brain network connectivity. By adopting a multidimensional assessment method, this study can reveal the brain function mechanism and the cognitive effects of traditional Chinese mind–body exercise on children with learning difficulties and provide new theoretical and practical bases for the fields of education and neuroscience. In addition, this study is significantly innovative because it is the first time that traditional Chinese mind–body exercises are applied to the intervention of children with learning difficulties, and their long-term effects on brain connectivity and cognitive functions are systematically assessed. This innovation not only enriches the theoretical system of intervention strategies for learning difficulties but also provides a feasible non-pharmacological intervention programmed for schools and families, which has strong practical significance. If this intervention model proves to be effective, it will have a profound impact on the education and mental health of children with learning difficulties and promote the development of personalized education and brain health intervention.

## 2. Research Methods

### 2.1. Participants

The study recruited 60 healthy children aged 9–11 years from ordinary schools in northern urban areas who were identified as having learning difficulties. Learning difficulties were defined using the following criteria: average scores in primary subjects (Chinese and Mathematics) ranking in the bottom 10th percentile of their respective class; performance in at least one specialized course below 60%; an Intelligence Quotient (IQ) > 70; and a score below 65 on the Pupil Rating Scale Revised Screening for Learning Disabilities (PRS). Exclusion criteria included children with physical activity impairments, diagnosed developmental disorders such as Attention Deficit Hyperactivity Disorder or Autism Spectrum Disorder, and other pediatric conditions such as epilepsy, severe visual or auditory impairments, or chronic illnesses. Ethical approval for this study was granted by the Ethics Committee of Beijing Normal University, and all procedures were conducted in accordance with the Declaration of Helsinki. Informed consent was obtained from the guardians of all participants.

### 2.2. Study Design

This study employed a randomized controlled design. A total of 80 participants were initially randomized into an intervention group (60 participants) and a control group (20 participants). The intervention group was further subdivided into three groups (20 participants each) based on the intervention type:

- Tai Chi group (TC).
- Baduanjin group (BD).
- Health Qigong Yijinjing group (YJJ).

**Figure 1:**
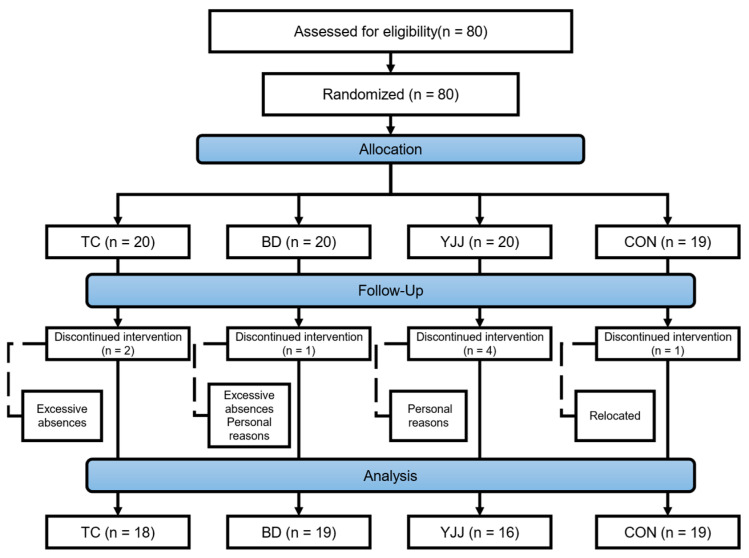
Flow chart for participant enrollment, allocation, and follow-up. TC, Tai Chi group; BD, Baduanjin group; YJJ, Yijinjing group; CON, control group.

Participants were randomly assigned to each experimental group in a 1:1 ratio of males to females, thereby achieving an equal gender distribution. Due to personal reasons or absenteeism, 7 participants withdrew from the intervention groups, and 1 participant from the control group was lost to follow-up due to relocation. The final sample consisted of 18 participants in the TC group, 19 in the BD group, 16 in the YJJ group, and 19 in the control group (CON). The participant allocation and retention flow are depicted in Figure 1.

Before the formal intervention, a total of three 30 min classes were arranged within one week to familiarize the participants in the intervention groups with the Chinese traditional mind—body exercises and teach them the key movement essentials. The intervention groups participated in Chinese traditional mind–body exercises for 12 weeks, with sessions occurring three times a week for 45 min per session. The control group did not receive any specialized mind–body intervention but maintained their regular physical education curriculum, which included activities such as rope skipping, ball throwing, and beanbag tossing. During the intervention period, the control group followed their usual routines without additional training.

### 2.3. Intervention Protocols

1. Tai Chi: The Tai Chi intervention used a simplified form of Yang-style Tai Chi adapted from a six-movement sequence endorsed by the National Sports Administration. This sequence included Opening Posture, Wave Hands Like Clouds, Double Whip, White Crane Spreads Its Wings, Cross Hands, and Closing Posture. Training began with demonstrations and detailed instruction by certified trainers. Participants were required to mimic the movements under supervision, focusing on posture and breathing techniques. Those who did not meet performance criteria were provided additional instruction and reassessment.
2. Baduanjin: The Baduanjin intervention followed the 2003 standardized “Health Qigong Baduanjin” form developed by the National Sports Administration. This practice consists of eight continuous, coordinated movements characterized by slow, controlled motions. Certified instructors demonstrated and monitored all movements, ensuring consistency with the standardized form. The training regimen mirrored the Tai Chi protocol in terms of structure and supervision.
3. Health Qigong Yijinjing: The Yijinjing intervention was based on the 2003 standardized “Health Qigong Yijinjing”. Six movements were selected to accommodate the study population: First Offering to Buddha, Second Offering to Buddha, Third Offering to Buddha, Spreading Wings, Nine Ghosts Drawing Swords, and Forward Bend. Training included demonstrations, movement correction, and safety monitoring, facilitated by a certified instructor and an assistant to ensure proper execution.

### 2.4. Measurement Tools

1. Academic Performance Ranking (APR)
  Academic performance in Chinese and Mathematics was measured through class ranking percentiles. Rankings were provided by homeroom teachers and included average scores for these two subjects. To ensure privacy, all scores were anonymized using participant identification numbers, protecting individual information confidentiality.
2. Pupil Rating Scale (PRS)
  The PRS, developed by H.R. Myklebust in 1981, includes verbal and non-verbal subscales and evaluates five behavioral domains: auditory comprehension and memory, language, temporal and spatial judgment, motor coordination, and social behavior. The scope of the scale is from 3 to 15 years of age. The scale is assessed by trained classroom teachers in each class. The Chinese version of this scale demonstrates excellent internal consistency (Cronbach’s α = 0.85)
3. Behavior Rating Inventory of Executive Function (BRIEF)
  The parent-reported version of the BRIEF, developed by Gioia et al. (2000), was used to assess executive functioning. It includes two indices, the Behavioral Regulation Index (BRI) and Metacognition Index (MI), further subdivided into eight domains: inhibition, shifting, emotional control, initiation, working memory, planning, organization, and monitoring. The scale comprises 86 items, rated on a three-point Likert scale, with higher scores indicating greater executive function deficits. The Chinese version of this scale demonstrates excellent internal consistency (Cronbach’s α = 0.86)

### 2.5. Neuroimaging Data Acquisition

**Figure 2:**
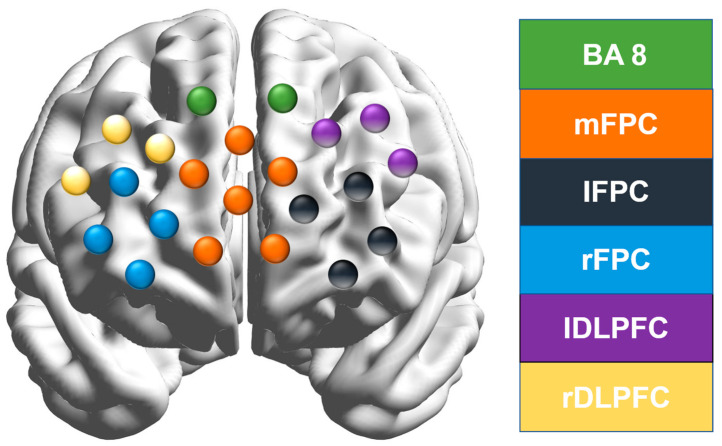
Six regions of interest in the prefrontal cortex.

Functional near-infrared spectroscopy (fNIRS) was used to measure changes in prefrontal cortex connectivity. The NIRStar system (nirX, Berlin, Germany), equipped with eight light sources and eight detectors forming 22 channels, was employed. The device operates at wavelengths of 695 nm and 830 nm, with a fixed emitter-detector distance of 3 cm. Data were sampled at 7 Hz. A modified international 10–20 system was used to place sensors on a soft cap designed for children, targeting the prefrontal cortex. Changes in oxygenated hemoglobin (HbO) concentration were calculated using the modified Beer–Lambert law. A total of 10 min of resting-state prefrontal cortex connectivity data were collected. Measurements were taken 30 min after a resting period following the executive function assessment. This study delineates the prefrontal cortex of children’s brains into six regions of interest, defined as the right dorsolateral prefrontal cortex (rDLPFC), including channels CH17, CH18, and CH22; the left dorsolateral prefrontal cortex (lDLPFC), including channels CH14, CH15, and CH19; the right frontal pole cortex (rFPC), including channels CH4, CH8, CH9, and CH13; the left frontal pole cortex (lFPC), including channels CH1, CH5, CH6, and CH10; the medial prefrontal cortex (mFPC), including channels CH2, CH3, CH7, CH11, CH12, and CH16; and area BA8, including channels CH20 and CH21, as illustrated in Figure 2. 

### 2.6. Brain Connectivity Analysis

Effective connectivity between brain regions was computed using the HERMES toolbox in MATLAB R2023a. Granger causality analysis was applied to estimate the directional connectivity. An autoregressive model was adopted, and the order of the model was estimated and calculated by the toolbox based on the Akaike Information Criterion and Bayesian Information Criterion. Eventually, the calculated value of the model order was 12. Effective connectivity strength between channels was represented using EC values. The analysis of effective connectivity is based on regions of interest. We calculated the differences in effective connectivity before and after the intervention and compared these differences across different groups.

### 2.7. Statistical Analysis

Outlier detection was conducted using boxplots, and normality was assessed using the Shapiro–Wilk test. As the data did not meet normality assumptions, non-parametric statistical methods were employed. The Kruskal–Wallis test was used for group comparisons, with post hoc multiple comparisons adjusted using the Holm–Bonferroni correction. The effective connectivity’s *p*-values were adjusted for multiple comparisons using the False Discovery Rate (FDR) correction. Spearman’s correlation analysis was used to explore the correlations between effective connectivity changes and behavioral changes. Statistical analyses were performed using SPSS 26.0 (IBM, Armonk, NY, USA), with *p* < 0.05 considered statistically significant.

## 3. Results

### 3.1. Baseline Data

The baseline data are presented in Table 1. The comparison results indicate that there were no significant differences in baseline data among the groups.

### 3.2. Post-Intervention Comparisons

As shown in Table 2, the results demonstrated significant differences in Pupil Rating Scale (PRS) scores among the groups (*p* = 0.000). Post hoc comparisons revealed that all three intervention groups had higher PRS scores than the control group (CON) (*p* = 0.041; *p* = 0.013; *p* = 0.000), with the Tai Chi group (TC) showing significantly higher scores than the Baduanjin group (BD) (*p* = 0.005).

Significant differences were also observed in Academic Performance Ranking (APR) scores among the groups (*p* = 0.010). Post hoc analysis indicated that all three intervention groups had higher APR scores compared to the CON group (*p* = 0.010; *p* = 0.006; *p* = 0.004).

For Inhibition scores, significant differences were found between the groups (*p* = 0.017). Post hoc comparisons showed that the TC group had significantly lower scores compared to the BD group and the CON group (*p* = 0.007; *p* = 0.004).

Finally, the Working Memory scores showed significant differences among the groups (*p* = 0.000). Post hoc comparisons indicated that the TC group had significantly lower scores compared to the BD group, YJJ group, and CON group (*p* = 0.001; *p* = 0.000; *p* = 0.000).

### 3.3. Effective Connectivity

**Figure 3:**
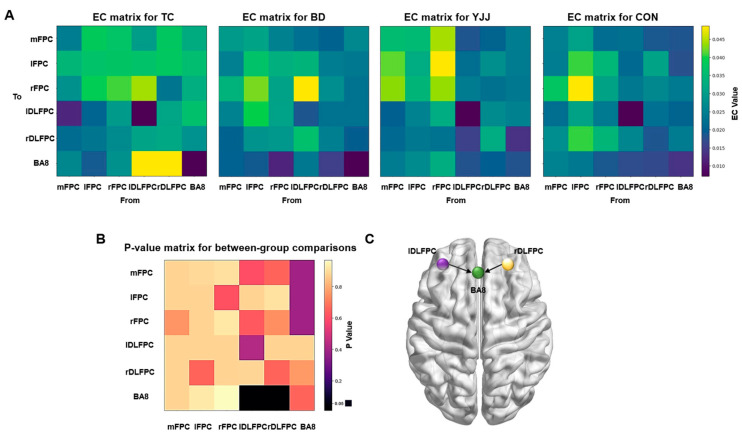
Comparison of pre- and post-intervention effective connectivity differences among groups. (**A**) Effective connectivity (EC) matrices of different intervention groups. (**B**) *p*-value matrix for inter-group comparisons. (**C**) Effective connectivity with significant inter-group differences.

Figure 3 illustrates the significant group differences in effective connectivity changes. Specifically, the connectivity difference from the left DLPFC to BA8 and the right DLPFC to BA8 showed significant group effects (P_FDR_ = 0.001; P_FDR_ = 0.000). Post hoc comparisons revealed that the TC group exhibited significantly higher effective connectivity (EC) changes from the left DLPFC to BA8 (*p* = 0.001; *p* = 0.007; *p* = 0.017) and from the right DLPFC to BA8 (*p* = 0.011; *p* = 0.0024; *p* = 0.011) compared to the BD group, YJJ group, and CON group.

Intra-group comparisons showed that, compared with pre-intervention, the EC values from the left DLPFC to BA8 in the TC group increased significantly (0.011 vs. 0.058, *p* = 0.000), and the EC values from the right DLPFC to BA8 in the TC group also increased significantly (0.008 vs. 0.062, *p* = 0.000). In the BD group, the EC values from the left DLPFC to rPFC increased significantly (0.006 vs. 0.053, *p* = 0.047). However, the changes in EC values in the BD group did not show significant inter-group differences.

In the correlation analysis between effective connectivity changes and behavioral changes presented in Table 3, only the correlation between Δ rDLPFC to BA8 and Δ APR was significant (r = 0.235, *p* = 0.047).

## 4. Discussion

### 4.1. Post-Intervention Effects on Learning Ability and Executive Functioning in Children with LDs

#### 4.1.1. Pupil Rating Scale

The results of this study indicated significant differences in PRS scores across the groups, with all three intervention groups—Tai Chi, Baduanjin, and Yijinjing—showing significantly higher PRS scores than the control group (CON). Specifically, the Tai Chi group achieved the highest PRS scores, suggesting that Tai Chi, along with Baduanjin and Yijinjing, can enhance learning ability, as reflected in the PRS scores. These findings align with Chen’s research, which demonstrated that mind–body exercises can positively impact children’s attention and cognitive engagement (Chen et al., 2024). The superior performance of the Tai Chi group in this study may be attributed to its slow, meditative nature, which enhances concentration and mind–body connection, promoting greater cognitive improvement (Shen et al., 2021). This improvement in PRS scores likely reflects enhanced self-regulation and sustained attention, crucial components for learning. Thus, Tai Chi seems to offer significant benefits in cognitive and attentional development, particularly in comparison to the other two mind–body exercises. The implications of these findings are significant for educational settings. By integrating Tai Chi, Baduanjin, and Yijinjing into school curricula, educators may be able to foster an environment that supports improved focus and cognitive engagement, leading to better academic outcomes. Moreover, these exercises may serve as effective interventions to support children with learning difficulties or attention deficits, providing them with a physical and mental outlet to enhance focus and concentration (Feng et al., 2024). These results suggest that incorporating mind–body exercises like Tai Chi, Baduanjin, and Yijinjing into daily routines could contribute to improving students’ ability to handle academic pressures, enhance cognitive flexibility, and manage stress more effectively (Liu et al., 2024).

#### 4.1.2. Academic Performance Ranking (APR)

This study found significant differences in APR scores across the groups, with all three intervention groups scoring higher than the control group. This suggests that traditional Chinese mind–body exercises can effectively enhance academic performance. While the differences among the intervention groups did not reach statistical significance, the Yijinjing group had the highest median APR score, indicating a potential stronger association with academic performance. This is consistent with Anzeneder’s study, which showed that regular physical exercise improves attention and stress management, thereby positively affecting academic outcomes (Anzeneder et al., 2023). The improvement in APR scores may result from the combined effects of physical activity and mental training, which promote neuroplasticity and executive function (St-Louis-Deschênes & Ellemberg, 2013). These findings further support the positive impact of traditional Chinese exercises on children’s academic achievement, particularly in areas such as concentration, information processing, and academic stress management. Furthermore, research suggests that engaging in activities that involve both the body and mind, such as Tai Chi and Yijinjing, can enhance cognitive flexibility and memory, both of which are crucial for academic success (G. Li et al., 2024). The holistic nature of these exercises, which integrate mindfulness, physical activity, and breath control, appears to foster an environment conducive to improved learning outcomes. Studies have also shown that regular practice of such exercises helps in reducing anxiety and increasing resilience, which are important for academic performance, especially under stressful conditions such as exams or deadlines. This combination of cognitive and emotional regulation may be a key factor in the academic improvements observed in the intervention groups (Solianik et al., 2022).

#### 4.1.3. Inhibition

Regarding the Inhibition indicator, the Tai Chi group scored significantly lower than the Baduanjin and control groups, with lower scores indicating better inhibitory control. Inhibitory control is a key component of executive function, enabling individuals to suppress impulsive responses and focus on goal-directed tasks. This result underscores Tai Chi’s effectiveness in improving inhibitory control. These findings align with Lin’s research, which found that Tai Chi enhances prefrontal cortex activity and executive function (Lin et al., 2022). In contrast, Baduanjin and Yijinjing may not have emphasized meditative focus to the same extent as Tai Chi, possibly leading to more limited improvements in inhibitory control. This study highlights the distinct benefits of Tai Chi in promoting self-regulation and inhibitory control, suggesting that this exercise may be particularly beneficial in developing these specific executive functions. Additionally, other studies have indicated that exercises such as Tai Chi, which require both mental and physical focus, can improve emotional regulation, enabling individuals to better control their impulses in emotionally charged situations (Winser et al., 2022). Moreover, strengthening inhibitory control through Tai Chi can contribute to improved task persistence, self-discipline, and decision-making, which are essential skills for students to succeed in both academic and personal aspects of their lives. The positive effects on inhibitory control may also transfer to other areas of life, such as improved self-regulation of behaviors and a reduced likelihood of engaging in risk-taking behaviors (X. Li et al., 2022).

#### 4.1.4. Working Memory

In terms of Working Memory, the results showed significant differences across the groups, with the control group scoring the highest, followed by the Yijinjing and Baduanjin groups, and the Tai Chi group scoring the lowest. Interestingly, the Tai Chi group scored significantly lower than all other groups, including the control group. This finding contradicts previous research, which generally suggests that traditional Chinese exercises enhance working memory. One possible explanation is that the physical and cognitive demands of Tai Chi may have temporarily increased the cognitive load on working memory for children with learning difficulties (Brocken et al., 2016). This is consistent with McNeill’s research, which indicates that while physical and mental exercise can improve certain executive functions, it may overload cognitive resources, especially for younger or cognitively challenged individuals. This study provides a novel perspective, suggesting that the cognitive improvements induced by traditional exercises can be complex and dynamic (McNeill et al., 2018). While Tai Chi excels in enhancing attention and inhibitory control, it may require a longer intervention period or further adaptation to fully support improvements in working memory.

In summary, the results of this study indicate that traditional Chinese mind–body exercises can significantly improve learning ability, executive functioning, and academic performance in children with learning difficulties. The effects of the three interventions varied across different cognitive domains: Tai Chi was particularly effective in enhancing attention and inhibitory control, while Yijinjing showed potential benefits for academic performance and working memory. These findings highlight the unique strengths of each exercise in targeting specific cognitive functions. They provide valuable insights for educators, therapists, and parents in designing tailored intervention programs for children with learning difficulties. Additionally, these results emphasize the need for future research exploring the long-term effects of these exercises and the underlying neural mechanisms driving cognitive improvements.

### 4.2. Post-Intervention Effects on Brain Function in Children with LDs

#### 4.2.1. Effective Connectivity and Pupil Rating Scale

In our study, we observed that the changes in effective connectivity from the left DLPFC (dorsolateral prefrontal cortex) to BA8, as well as from the right DLPFC to BA8, were significantly greater in the Tai Chi (TC) group compared to the Baduanjin (BD), Yijinjing (YJJ), and control (CON) groups. When combined with the results of the PRS scores, the TC group demonstrated significantly higher PRS scores than the other intervention groups, indicating that Tai Chi was particularly effective in enhancing the learning abilities of children with learning difficulties. The observed enhancement in connectivity between DLPFC and BA8 may have played a central role in this process. The DLPFC is known as a core region for executive functioning, responsible for regulating attention, working memory, and task management, while BA8 is associated with goal-oriented behavior and the maintenance of attention (Nejati & Estaji, 2024). The enhanced connectivity suggests that Tai Chi strengthens attentional regulation and cognitive engagement by improving the synchronization of these brain regions. This finding aligns with previous research, reinforcing the notion that Tai Chi promotes both cognitive and academic performance. Tai Chi’s slow, meditative movements may enhance the functional connectivity between brain regions, fostering improved focus and sustained attention in children with learning difficulties (Yao et al., 2019).

Additionally, our study introduces the novel finding that the enhanced connectivity from DLPFC to BA8 could serve as one of the neural mechanisms underpinning the elevated PRS scores. This suggests that Tai Chi indirectly enhances attentional regulation and self-management by improving brain functional connectivity, which, in turn, supports the significant improvement of learning abilities in children with learning difficulties (Andoh, 2020).

#### 4.2.2. Effective Connectivity and Academic Performance Ranking (APR)

This study also found that all three intervention groups showed significantly higher APR scores than the control group, with the YJJ group achieving the highest median APR scores. This suggests that Yijinjing may have an advantage in improving academic performance. However, despite these findings, the Tai Chi group demonstrated more pronounced changes in brain functional connectivity. This implies that different mind–body exercises may influence academic performance through distinct neural mechanisms. The improved APR could be linked to enhanced connectivity from the DLPFC to BA8. The DLPFC’s ability to modulate executive functions is crucial not only for attention and memory but also for managing academic stress (Nejati et al., 2023). Previous studies have shown that exercise can enhance children’s academic performance by reducing stress and improving attention (Tomporowski et al., 2015). The observed academic performance rankings are likely linked to brain connectivity changes, with different mind–body exercises affecting neural mechanisms in distinct ways. The Yijinjing group showed improved APR scores, potentially due to enhanced connectivity between the DLPFC and BA8, which supports executive functions like attention, memory, and self-control, helping students manage academic tasks more efficiently (Orth et al., 2023). On the other hand, the Tai Chi group demonstrated changes in brain connectivity related to inhibitory control and stress regulation, which are crucial for academic success. Tai Chi’s focus on mindfulness and stress reduction may improve emotional regulation, helping students manage academic pressure. These findings suggest that both types of exercise influence brain connectivity through different neural pathways, contributing to academic performance in complementary ways by enhancing both cognitive and emotional functions (Wei et al., 2021).

#### 4.2.3. Effective Connectivity and Inhibition

The Tai Chi group outperformed all other groups in terms of inhibitory control, as indicated by significantly better inhibition scores (lower scores reflecting better inhibitory control). This result is consistent with the enhanced DLPFC to BA8 connectivity, as both of these brain regions are involved in inhibitory control and goal-directed behavior. The improved connectivity likely contributed to enhanced focus and impulse control when faced with distractions. This finding aligns with previous studies showing that Tai Chi can improve inhibitory control by boosting prefrontal cortex activity. The present study extends this understanding by identifying that the enhanced connectivity between the DLPFC and BA8 may be a key neural mechanism underlying the improvement in inhibitory control (X. Li et al., 2022). Furthermore, Tai Chi outperformed both the Baduanjin and Yijinjing groups in terms of inhibitory capacity, likely due to its unique movement patterns and practice characteristics. Tai Chi emphasizes slow, deliberate movements, meditation, and concentration, all of which contribute to strengthening the ability to allocate attention and self-regulate. These features may directly enhance inhibitory control by improving functional connectivity within the DLPFC (Converse et al., 2014).

#### 4.2.4. Effective Connectivity and Working Memory

Interestingly, the Tai Chi group scored significantly lower on working memory tasks compared to the other groups, including the control group. While this finding contrasts with previous studies, it can be explained through the analysis of functional connectivity: the marked enhancement in connectivity from DLPFC to BA8 may have preferentially facilitated certain executive functions, such as inhibitory control and attention. In contrast, improvements in working memory might require a longer duration of intervention or practice. Previous studies have suggested that mind–body exercises may place an additional cognitive load on working memory, especially in children with learning difficulties, as these exercises often involve complex body movements that require cognitive resources. Our results support the hypothesis that Tai Chi may lead to a temporary reallocation of cognitive resources, which could limit the short-term enhancement of working memory (Xu et al., 2023). Moreover, working memory and other executive functions are dynamically interconnected, and Tai Chi may have temporarily prioritized the enhancement of attention and inhibitory control, while working memory improvements may require more prolonged practice (Sarabzadeh et al., 2019). Future studies could explore whether longer interventions could address this limitation and further substantiate the potential benefits of Tai Chi on working memory.

Research limitations and recommendations for guidance: This study provides valuable insights into the effects of traditional Chinese mind–body exercises on children with learning difficulties, but several limitations should be acknowledged. The small sample size, short duration of the intervention, and lack of a diverse sample limit the generalizability of the findings. Additionally, other potential confounding factors, such as home environment and individual differences in motivation, were not accounted for. Future research should aim to expand the sample size to include a more diverse population and implement longitudinal studies to assess the long-term impact of these exercises on executive functions, cognitive development, and academic performance. Exploring the underlying mechanisms through neuroimaging or electrophysiological methods would further our understanding, and developing clear guidelines for implementing these interventions in educational settings would be beneficial for supporting children with learning difficulties.

## 5. Conclusions

This study demonstrates that traditional Chinese mind–body exercises, such as Tai Chi, Baduanjin, and Yijinjing, have a significant positive impact on academic performance and executive function in children with learning difficulties. Although differences between the intervention groups did not reach statistical significance, the Yijinjing group showed the highest Academic Performance Ranking (APR), suggesting a potentially stronger association with academic achievement. Notably, Tai Chi significantly improved inhibitory control, highlighting its particular effectiveness in enhancing executive function. Furthermore, this study is the first to systematically assess the long-term effects of traditional mind–body exercises on brain connectivity and cognitive function in children with learning difficulties, providing strong theoretical and practical evidence for future educational interventions. These findings suggest that incorporating these exercises into educational programs may enhance not only cognitive abilities but also emotional regulation, resilience, and stress management. If these intervention models are further validated through larger, more diverse studies, they could have a profound impact on personalized education strategies and brain health interventions, offering a promising non-pharmacological approach to supporting children with learning difficulties.

## Figures and Tables

**Table 1:** Comparison of baseline data between groups.

	TC	BD	YJJ	CON	*p*
Age (year)	10.00 (1.00)	9.00 (1.00)	10.00 (2.00)	10.00 (2.00)	0.370
Body Height (cm)	141.66 (10.89)	136.70 (3.72)	133.31 (9.10)	142.04 (10.95)	0.263
Body Weight (kg)	41.65 (13.92)	36.60 (5.81)	31.63 (11.88)	42.05 (13.38)	0.263
PRS	51.00 (12.00)	55.00 (17.50)	46.00 (18.50)	47.00 (17.00)	0.225
APR	8.00 (2.75)	7.00 (3.00)	8.00 (1.25)	8.00 (2.50)	0.988
Inhibition	17.00 (5.50)	16.00 (5.00)	18.00 (7.50)	16.00 (7.00)	0.324
Shifting	10.00 (2.00)	10.00 (2.00)	10.00 (3.25)	10.00 (3.00)	0.519
Emotional control	16.00 (3.75)	17.00 (2.50)	17.00 (3.00)	17.00 (4.00)	0.536
Initiation	14.00 (0.00)	14.00 (1.00)	14.00 (1.25)	14.00 (1.50)	0.882
Working memory	23.00 (4.00)	21.00 (3.50)	20.00 (5.00)	22.00 (5.00)	0.101
Planning	26.00 (4.00)	26.00 (4.50)	27.00 (2.25)	25.00 (4.00)	0.781
Organization	12.00 (3.75)	11.00 (3.50)	11.00 (3.00)	10.00 (3.00)	0.940
Monitoring	16.00 (1.75)	16.00 (3.50)	16.50 (3.00)	14.00 (3.50)	0.108

Note: Abbreviations: TC, Tai Chi group; BD, Baduanjin group; YJJ, Yijinjing group; CON, control group; PRS, Pupil Rating Scale Revised Screening for Learning Disabilities; APR, Academic Performance Ranking.

**Table 2:** Comparison of indicators between groups after the intervention.

	TC	BD	YJJ	CON	*p*	*η*^2^
PRS	76.00 (3.50)	71.00 (9.50) ^a^	73.50 (6.50)	65.00 (4.50) ^abc^	0.000	0.334
APR	15.50 (8.50)	16.00 (6.50)	17.00 (7.50)	11.00 (5.50) ^abc^	0.010	0.160
Inhibition	7.00 (3.00)	11.00 (4.00) ^a^	10.00 (3.00)	11.00 (4.00) ^a^	0.017	0.144
Shifting	10.00 (1.75)	10.00 (2.50)	11.00 (2.00)	10.00 (2.00)	0.193	0.067
Emotional control	13.00 (2.75)	13.00 (3.00)	12.50 (3.00)	12.00 (1.50)	0.599	0.026
Initiation	14.00 (5.75)	15.00 (3.00)	14.50 (2.25)	15.00 (3.00)	0.505	0.033
Working memory	10.00 (1.75)	15.00 (2.00) ^a^	15.50 (2.25) ^a^	16.00 (2.00) ^a^	0.000	0.390
Planning	17.00 (6.75)	17.00 (7.00)	17.00 (3.25)	17.00 (6.50)	0.836	0.012
Organization	10.50 (4.00)	12.00 (3.50)	11.00 (2.25)	10.00 (2.50)	0.810	0.014
Monitoring	16.00 (1.75)	16.00 (3.50)	16.50 (3.00)	14.00 (3.50)	0.108	0.086

Note: Abbreviations: TC, Tai Chi group; BD, Baduanjin group; YJJ, Yijinjing group; CON, control group; PRS, Pupil Rating Scale; APR, Academic Performance Ranking. ^a^ indicates significant difference compared to TC group; ^b^ indicates significant difference compared to BD group; ^c^ indicates significant difference compared to YJJ group.

**Table 3:** Correlation analysis between effective connectivity changes and behavioral changes.

	Δ lDLPFC to BA8	Δ rDLPFC to BA8
Δ PRS	0.093 (0.425)	0.145 (0.226)
Δ APR	−0.057 (0.634)	0.235 (0.047)
Δ Inhibition	0.028 (0.815)	0.029 (0.812)
Δ Working memory	0.221 (0.062)	0.071 (0.553)

The data are presented in the form of correlation coefficients and *p*-values.

## Data Availability

Data are contained within the article.
